# Prevalence of cataract in adult Down's syndrome patients aged 28 to 83 years

**DOI:** 10.1186/1745-0179-3-26

**Published:** 2007-11-22

**Authors:** Basant K Puri, Iqbal Singh

**Affiliations:** 1 MRI Unit, Imaging Sciences Department, MRC Clinical Sciences Centre, Imperial College London, Hammersmith Hospital, London W12 0HS, UK; 2 Mental Health Unit, Hillingdon Hospital, Pield Heath Road, Uxbridge, Middlesex UB8 3NN, UK

## Abstract

**Background:**

Age-related cataract is the major cause of blindness in humans throughout the world. The majority of previous studies of cataract in Down's syndrome (which usually results from trisomy 21) have reported that the prevalence of this ocular abnormality is higher for a given age range than in the general population. The objective of the present study was to study the prevalence of cataract in a well-defined population of adults with Down's syndrome.

**Methods:**

An in-patient population of 68 adults (35 males and 33 females) with Down's syndrome, aged between 28.9 and 83.3 years, underwent ophthalmological examination for the presence of cataracts.

**Results:**

Overall, the prevalence of cataract was 16.2%, with no significant difference in the prevalence between males (17.1%) and females (15.2%). In those aged between 45 and 64 years, the prevalence was 16.7%, rising in those aged between 65 and 75 years to 28.6%.

**Conclusion:**

Compared with the general population, the prevalence of cataract in Down's syndrome was raised in those aged 45 to 64, but not in those aged 65 to 75 years; the latter might be a function of the relatively small number of patients in this age group. The increased prevalence of cataract found in those in the 45- to 64-year-old age group may be the result of increased levels of the copper- and zinc-containing superoxide dismutase enzyme (CuZnSOD), in turn resulting from the location of the associated five exons of *SOD1 *on chromosome 21. These elevated levels of superoxide dismutase may give rise to increased levels of reactive species, including hydrogen peroxide and hydroxyl radicals, which may increase the risk of cataractogenesis. It is suggested that nutritional supplementation with antioxidants may therefore help reduce the prevalence of cataract in Down's syndrome.

## Introduction

Age-related cataract is the major cause of blindness in humans throughout the world. In England and the United States the prevalence of cataract in the general population aged 45 to 64 years is between 2 and 8%, rising to between 21 and 39% in the 65 to 75 year age group, and to 65% in those aged 85 years and over [1-3].

The majority of previous studies of cataract in Down's syndrome have reported that the prevalence of this ocular abnormality is higher for a given age range than in the general population (see Table 1). The present study examined the prevalence of cataract in adult Down's syndrome.

**Table 1:** Studies of the prevalence of cataract in Down's syndrome

Study	No. of subjects	Age of subjects (years)	Percentage with cataract
Lowe 1949 [11]	67	< 60	87
Skeller & Øster 1951 [12]	80	< 58	46
Cullen & Butler 1963 [13]	143	2–53	15
Chutko 1965 [14]	100	< 18	20
Rochels et al. 1977 [15]	1047	< 23	2
Gnad & Rett 1979 [16]	420	< 14	55
Jaeger 1980 [17]	75	15–68	55
Walsh 1981 [18]	91	5–60	17
Shapiro & France 1985 [19]	53	7–36	13
Riise 1986 [20]	123	< 66	59
Hestnes et al. 1991 [21]	30	21–72	50
Da Cunha & Moreira 1996 [22]	152	< 18	13
Present study	68	28–84	16

## Methods

All adults with Down's syndrome in the catchment area of Leavesden Hospital, a large hospital for individuals with learning disability in Hertfordshire, England, were examined in detail clinically and underwent full ophthalmological examination for the presence of cataracts. They included in-patients, day-patients, and those in the community.

Statistical analyses were carried out the SPSS for Windows program (Chicago, USA).

## Results

The total number of adults with Down's syndrome was 68, consisting of 35 males and 33 females, ranging in age from 28.9 to 83.3 years (mean 54.1 years, standard deviation (sd) 11.9 years). Eleven individuals were identified as having an ophthalmological diagnosis of cataract, representing a prevalence of 16.2% of the population studied, and there was no significant difference between males (6/35) and females (5/33) (*p *= 0.82). The mean age of those with a history of cataract was 55.3 (sd 9.0) years compared with 53.8 (sd 12.4) years for those without such a history; this difference was not significant (*p *= 0.70). The two groups (those with and those without cataract) were also matched for sex (male: female = 6:5 versus 29:28, respectively; χ^2 ^= 0.011; *df *= 1; *p *> 0.9).

For those aged between 45 and 64 years, the prevalence was 16.7% (7/42), while for those in the age range 65 to 75 years, the prevalence was 28.6% (2/7).

## Discussion

Ocular abnormalities are relatively common in individuals with mental retardation, particularly those with Down's syndrome [4]. In the present study, the prevalence of cataract found in adults with Down's syndrome is consistent with that reported in several previous studies (Table 1). For those aged between 45 and 64 years, the prevalence of cataract in Down's syndrome (16.7%) was significantly greater than that in the general population of England and the United States (2 to 8%). Indeed, Das et al [5] found a prevalence of age-related cataract, aphakia or pseudophakia of zero in a European sample aged between 40 and 49 years, and of 6% (standard error 4.3%) in those aged between 50 and 59 years. For those aged between 65 and 75 years, the prevalence in Down's syndrome (28.6%) was not significantly different from that in the general population (21 to 39%).

Our finding that the prevalence of cataract in those aged between 65 and 75 years did not differ from the prevalence in the general population may be a function of the relatively small number of cases (seven) in our sample in this age group. It is important to consider the possible cause for the impression of an increased rate of cataract in the 45- to 64-year-old age group. The general consensus is that cataracts are usually the result of free radical damage. There is chronic exposure of the ocular surface to oxidative stress, for example from atmospheric oxygen and exposure to ultraviolet light (in sunlight). However, these factors affect Down's syndrome individuals and non-Down's syndrome individuals alike. The question then becomes: is there any evidence that Down's syndrome individuals have greater exposure to free radicals?

Dickinson and Singh have proposed that in Down's syndrome there is increased activity of superoxide dismutase, resulting in overproduction of hydrogen peroxide and hydroxyl free radicals which in turn compromise cellular functioning [6]. Individuals with Down's syndrome typically have trisomy 21; some have only additional portions of chromosome 21, while others show mosaicism [6]. The result is an increased production of many of the proteins encoded by genes on this chromosome, including the copper- and zinc-containing superoxide dismutase enzyme (CuZnSOD); indeed, one-and-a-half times the normal CuZnSOD activity is found in cells from trisomy 21 individuals [7]. It is possible that some of the characteristic features of Down's syndrome may be related to increased levels of CuZnSOD. Not only do transgenic mice over-expressing human CuZnSOD show many of the neurological and neurochemical features that are seen in human Down's syndrome, but they also have ocular lenses which are more susceptible to photochemical damage *in vitro *[7,8]. Elevated levels of CuZnSOD may exacerbate the pro-oxidant effects of this enzyme; in the presence of millimolar concentrations of hydrogen peroxide, CuZnSOD can generate hydroxyl free radicals, and can catalyze the oxidation of azide, urate and nitrite (to the reactive nitrogen species NO_2_^•^) [6,7]. Down's syndrome individuals have indeed been found to have increased erythrocyte superoxide dismutase activity compared with control subjects [9], high levels of which, in non-Down's syndrome individuals, have been found to be associated with increased risk of cataract [10].

In light of the increased levels of superoxide dismutase (and therefore increased levels of hydrogen peroxide and other reactive species) associated with Down's syndrome, it seems possible that antioxidant therapy might be helpful in preventing cataractogenesis in this group of individuals. Indeed, Dickinson and Singh have previously suggested that vitamin E, and possibly vitamin C, may be used as a therapy in the treatment of the dementia associated with Down's syndrome, since they may modify the damage caused by excessive hydrogen peroxide production [6].

## Conclusion

Compared with the general population, the prevalence of cataract in Down's syndrome was raised in those aged 45 to 64, but not in those aged 65 to 75 years. The increased prevalence of cataract may be the result of increased levels of CuZnSOD, in turn resulting from the location of the associated five exons of *SOD1 *on chromosome 21. These elevated levels of superoxide dismutase may give rise to increased levels of reactive species, including hydrogen peroxide and hydroxyl radicals, which may increase the risk of cataractogenesis. Therefore, nutritional supplementation with vitamin E, and possibly vitamin C, may help reduce the prevalence of cataract in Down's syndrome.

## Authors' contributions

BP and IS conceived of the study, and participated in its design and coordination.

BP carried out the statistical analyses.

Both authors read and approved the final manuscript.
